# Approval of Parent-Child Aggression as a Mediator of Intergenerational Child Abuse Risk: An Evaluation of Racial Differences

**DOI:** 10.1007/s10896-024-00799-5

**Published:** 2025-01-13

**Authors:** Casie H. Morgan, Christina M. Rodriguez, Doris F. Pu, Zoe O. Elkins

**Affiliations:** 1 Medical University of South Carolina, Charleston, SC, USA; 2 Old Dominion University, Norfolk, VA, USA; 3 Mount Sinai Services/NYC Health + Hospitals/Queens, Jamaica, NY, USA; 4 University of Alabama at Birmingham, Birmingham, AL, USA; 5 Department of Psychology, Old Dominion University, 250 Mills Godwin Life Sciences Building, Norfolk, VA 23529, USA

**Keywords:** Parenting, Race, Physical and psychological child abuse, Intergenerational transmission of violence, Cycle of violence

## Abstract

**Purpose:**

Personal history of parent-child aggression (PCA) can predict future parenting behavior, but some effects may differ between racial groups. Black parents in the U.S. are more likely to encounter discrimination and personally experience and approve of PCA, which may account for previously reported group differences. This study examined whether personal PCA history predicted later parental child abuse risk mediated by PCA approval across the transition to parenthood, and whether effects differed by race.

**Methods:**

Expectant parents (non-Hispanic White sample: 95 mothers with 86 fathers; Black sample: 94 mothers with 85 fathers) participated in a prospective longitudinal study, assessed prenatally and when children were age 6 mo., 18 mo., and four years. Personal history of PCA was assessed retrospectively, PCA approval was evaluated at each timepoint; and abuse risk was assessed as both theoretical abuse risk (an analog task at all timepoints) and actual PCA use (parents’ report at the final two timepoints).

**Results:**

Personal PCA history largely predicted PCA approval for Black parents but was inconsistent for White parents. Higher PCA approval predicted abuse risk for both groups but appears to be a more consistent mediator between personal PCA history and abuse risk for Black parents.

**Conclusions:**

Findings suggest PCA approval may perpetuate the PCA cycle but future work needs to consider differential effects by race, socioeconomic status, and age and identify factors that may account for such differences. Abuse preventions should be more intentionally culturally informed to enhance efficacy for communities of color.

Physical abuse—actual or threat of physical harm—and psychological abuse—aggression or omissions inducing fear that impacts emotional well-being and functioning—are prominent examples of child maltreatment (U.S. Department of Health & Human Services [DHHS], 2022). Despite mandatory reporting initiatives, child maltreatment is vastly underreported (Euser et al., 2015; Meinck et al., 2016; Sedlak et al., 2010), with some estimates from U.S. surveys indicating that rates of physical abuse alone are exponentially higher than those referred through official channels (Simon et al., 2018). Due to recognized underreporting, researchers and practitioners often instead evaluate a parent’s *child abuse risk*, a concept intended to gauge both the parenting beliefs and behaviors of a parent that increase their risk to engage in abusive behavior (e.g., Bavolek & Keene, 2001; Chaffin & Valle, 2003). The use of any form of parent-child aggression (PCA) increases parents’ likelihood to abuse (Afifi et al., 2017a; Gershoff & Grogan-Kaylor, 2016b). All types of PCA conceptually occur along a continuum, with common parenting discipline strategies (e.g., spanking and yelling) on one end of the continuum and physical and psychological abuse on the other end; PCA use that intensifies along this continuum can lead to abusive behavior (e.g., Durrant et al., 2009; Rodriguez et al., 2021). Parents’ child abuse risk can be estimated by parent report of aggressive behavior during parent-child conflict (Chan, 2012; Straus et al., 1998), parental physical discipline use (Afifi et al., 2017b; King et al., 2018; Rodriguez, 2010), as well as psychologically harmful discipline (Afifi et al., 2017a, b; Helwig et al., 2014).

Many distinct factors contribute to parents’ increased child abuse risk (e.g., Doidge et al., 2017; Maguire-Jack & Font, 2017; Rodriguez et al., 2018b), with intergenerational transmission theory underscoring the significance of a parent’s personal history of receiving PCA in increasing their child abuse risk (Greene et al., 2020). Standard intergenerational theories of child abuse suggest that those abused as a child are more likely to be abusive as a parent (Bartlett et al., 2017; Buisman et al., 2020; Greene et al., 2020). Through the influence of social learning, role modeling from one’s parents shapes the emergence of future parenting beliefs as well as behaviors (Martoccio et al., 2022), suggesting that parenting practices reflect what parents observed as children. Mothers with a greater personal history of physical punishment and verbal hostility are more likely to report spanking as their choice of discipline as well as beliefs that favor PCA use (Chung et al., 2009). Additionally, mothers’ and fathers’ personal history of both physical and psychological aggression relate to their stronger approval of PCA use across time (Rodriguez et al., 2018a), with similar findings of approval of PCA for those with a personal history of physical discipline (Deater-Deckard et al., 2003; Durrant et al., 2018). Endorsement of PCA use is a belief that increases parents’ child abuse risk, as evidenced in international research between positive perceptions of PCA and parental reports of harsh physical and non-physical discipline use by both mothers and fathers (Lansford et al., 2014). Meta-analytic findings support that approval of PCA is an attitude that contributes to parents’ greater physical child abuse risk (Camilo et al., 2020), suggesting PCA approval may be a prerequisite for parents’ use of both physical and psychological aggression (Combs-Orme & Cain, 2008; Rodriguez et al., 2021). Given the evidence demonstrating personal PCA history contributes to parents’ approval of PCA, which in turn relates to parents’ later use of PCA, targeting parental beliefs regarding approval of PCA as a discipline technique appears critical to decreasing parents’ child abuse risk.

The purpose of the current study was to investigate the potential differential role of PCA approval on parental child abuse risk focused on racial group differences. Previous research characterizes the link between PCA approval and PCA use as universal, suggesting that these relationships exist internationally (Lansford et al., 2014). Yet recent research has also observed racial differences between Black and White families when examining parents’ child abuse risk in the U.S. (Rodriguez et al., 2021; Silveira et al., 2021). Black children are more likely to experience harsh physical punishment (Gershoff et al., 2012; Finkelhor et al., 2019; Taillieu et al., 2014) and are more likely to witness physical violence and abuse in the home compared to other children (Crouch et al., 2000). Furthermore, although trends of harsh physical punishment use appear to be declining in the U.S., this trend is less notable among Black families compared to White families (Taillieu et al., 2014).

Thus, PCA appears more common in Black families, which would imply Black parents are more likely to possess greater personal history of childhood PCA compared to White parents. Intergenerational links between personal history of PCA and PCA approval (Durrant et al., 2018; Rodriguez et al., 2018a) may explain why Black parents thus demonstrate higher PCA approval (Chiocca, 2017; Su et al., 2018) as well as greater PCA use. Black parents evidence higher child abuse risk (Combs-Orme et al., 2000; Rodriguez et al., 2021), higher rates of harsh physical discipline use (Pratt et al., 2004; Silveira et al., 2021), and greater likelihood to spank their children compared to other groups (Berlin et al., 2009; Gershoff et al., 2012).

Notably, however, race itself is not an explanatory factor. Previously observed differences in child abuse risk between Black and White families may be attributable to several critical contextual factors. First, greater experience of racial discrimination is associated with parental physical punishment and maltreatment (Scott, 2017) and indirectly contributes to family violence among Black men, particularly for those with a personal history of experiencing physical PCA (Sutton et al., 2020). These connections to racism highlight the pressure Black parents may feel to quickly change their child’s behavior and increase compliance. Black parents may be socializing their children to understand the severe and unique social threats experienced by Black individuals, wherein parents strive to enforce compliance so that children anticipate and avoid those threats (Silveira et al., 2021). Therefore, Black parents may feel compelled to utilize harsh physical or psychological PCA from a more protective mindset (Patton, 2017; Silveira et al., 2021). Additionally, stress related to discrimination and structural disadvantages can magnify parents’ psychological distress (Gordon et al., 2016), which could ultimately contribute to greater implementation of physical and verbal aggression. Consistent with structural disadvantages, historical racial inequities associated with economic (Michener & Brower, 2020) and educational (Jeffrey, 2020) disparities may play a role as well. Research indicates that socioeconomically segregated neighborhoods can lead to higher concentrations of violence, higher rates of maltreatment victimization, and greater family discord (Leventhal & Dupéré, 2019). The racial segregation of these same neighborhoods can contribute to heightened levels of structural racism and discrimination which further impacts family well-being and parenting practices (Sampson, 2019). Yet, a recent study observed significant differences between Black and White parents in the relations between low educational attainment and endorsement of spanking: contrary to previous literature (e.g., McLoyd, 1998), White parents with lower educational attainment were found to be more likely to endorse spanking compared to Black parents (Schneider & Schenck-Fontaine, 2022).

Cultural traditions of PCA use may play a role as well (Gershoff & Grogan-Kaylor, 2016a), reflecting the potentially greater relevance of personal history of experiencing PCA in Black families. Black parents may adopt using PCA as a discipline tool as part of their family heritage because they experienced it more frequently themselves relative to their White peers, serving as their cultural frame of reference when parenting their own children (Patton, 2017; Silveira et al., 2021). Using a cultural parenting perspective, Patton and colleagues (2021) review the literature on race, physical punishment, and White supremacist structures and conclude that the parenting style of Black culture is characterized by approaches that approve the implementation of physical discipline in anticipation that community authority figures will implement physical violence against Black youth through adulthood. Recent research highlights the importance of explicitly understanding what may contribute to racial differences in parenting practices and abuse risk (Rodriguez et al., 2021), particularly because Black children are overrepresented in the child welfare system (Cénat et al., 2021). Better identification of what factors may underlie potential racial differences in child abuse risk could better inform the development of more culturally appropriate child abuse prevention programs.

## Current Study

The present study aimed to examine the role of PCA approval as a differential factor in how personal history of PCA contributes to parents’ child abuse risk (operationalized broadly as a theoretical construct that can be assessed even prior to parenthood as well as parents’ actual PCA use), directly comparing Black versus non-Hispanic White mothers and fathers. Prior research suggests PCA use is intergenerational and that approval of PCA is a precursor for its use. However, earlier work has not adequately examined whether these two risk factors (personal history and PCA approval) may differ between racial groups in contributing to parents’ abuse risk. Clarifying what contributes to racial differences can move the field from considering race itself as an explanatory variable given systemic racial inequities. Because child abuse is greatest amongst children younger than three (DHHS, 2022), and the focus of most abuse prevention programs target parents during the prenatal and perinatal period (e.g., Pajer et al., 2014), this study focused on the transition to parenthood. The current study examined whether greater PCA approval mediates the link between personal PCA history and child abuse risk across the transition to parenthood differently by race. For the following hypotheses, child abuse risk refers to both theoretical PCA risk and actual PCA use. Within this mediation analysis, two broad hypotheses were considered: (H1) Personal PCA history was expected to relate to PCA approval (H1a), PCA approval was expected to relate to greater child abuse risk (H1b), and greater personal experience of physical and psychological PCA was hypothesized to directly predict parents’ child abuse risk (H1c), with potentially stronger effects observed for Black parents (H1d). (H2) PCA approval was expected to partially mediate the association between personal PCA history and parents’ child abuse risk (H2a), but more strongly for Black parents than for White parents (H2b).

## Method

### Participants and Procedures

The current sample involved parents participating in a prospective longitudinal study monitoring the development of child abuse risk, the Following First Families (“Triple-F”) study. Recruited from ob/gyn and childbirth classes at local hospitals in a large Southeastern U.S. city, primiparous women and their male partners were enrolled during the last trimester of women’s pregnancy for a three-wave study. The Triple-F study oversampled mothers demonstrating sociodemographic risk, with 53.2% of mothers meeting at least one of the following criteria: (a) 150% below the poverty line; (b) receipt of public assistance; (c) high school education or less; (d) age 18 or younger. Mothers and fathers provided written informed consent independently and completed the study protocol in separate areas from each other on laptop computers with headphones. The university’s Institutional Review Board approved all study procedures.

At Time 1 (T1), 203 families, with 203 women and 151 of their male partners were enrolled during the last trimester of the mother’s pregnancy. Families were reassessed when their infant was 6 months old (± 2 weeks) for Time 2 (T2) and again when their toddler was 18 months old (± 3 weeks) for Time 3 (T3). A fourth wave (Time 4; T4) was later added to the Triple-F study, locating families who were still locally available when their children turned four (4 years, 0 months– 4 years, 6 months). By T2, two families were no longer eligible to continue because their baby died soon after childbirth, resulting in 186 families (186 mothers and 146 male partners) that continued in the study. By T3, one family had lost custody of their child, resulting in 180 families (180 mothers and 144 male partners) that participated in the study. By T4, an additional family had lost custody; 120 families (119 mothers and 93 male partners) participated in the study. The current investigation included the 360 parents in 189 families from Time 1 who self-identified as Black (*n*=179 mothers and fathers from 94 families) or non-Hispanic, non-biracial White (*n*=181 mothers and fathers from 95 families). Based on a medium effect size, an estimated ICC of .30, and power of .80 for the longitudinal study, a minimum sample size of 79 per gender was established.

At T1, the White subsample included 95 mothers and 86 fathers. The average age of this group was 30.13 (*SD*=4.69) years, with about half of households reporting an annual income between $70,000-$79,999 and 8.2% reporting receipt of public assistance. With respect to educational attainment: 5.3% high school or less; 15.8% some college or vocational training; 39.2% college degree; and 39.8% beyond college degree. The Black subsample of parents included 94 mothers and 85 fathers (age *M*=24.04, *SD*=5.96). About half of the Black subsample reported an annual income between $8,000-$12,999, with 58.9% reporting receipt of public assistance. With regard to educational attainment: 55.7% high school or less; 25.9% some college or vocational training; 8.2% college degree; and 10.1% beyond college degree.

## Measures

### Independent Variable: Personal PCA History

A retrospective report on the *Parent-Child Conflict Tactics Scales* (CTSPC; Straus et al., 1998) was used at Time 1 to assess parents’ experience of PCA, in which respondents reported on the frequency with which they had personally experienced 22 items during childhood. Each item is rated (and scored) as follows: 0=never happened (scored 0); 1=once (scored 1); 2=twice (scored 2); 3=3–5 times (scored 4); 4=6–10 times (scored 8); 5=11–20 times (scored 15); 6=more than 20 times (scored 25). The CTSPC includes 13 items on physical PCA of widely varying intensity (e.g., spanking to burning with a cigarette) and 5 items on psychological PCA (e.g., yelling to name-calling), with item ranges of 0–25. Summed across these weighted frequency counts for all 18 items, higher CTSPC Combined Assault History scores signify greater personal experience of PCA.

### Mediator: PCA Approval

The *Value of Corporal Punishment Scale* of the Adult Adolescent Parenting Inventory-2 (AAPI-2; Bavolek & Keene, 2001) was used at all four time points to assess parents’ approval of PCA as a discipline approach. Participants indicated their level of agreement with 11 items from 1 (strongly disagree) to 5 (strongly agree); summed across items, higher AAPI-2 Corporal Punishment scale total scores indicate stronger PCA Approval. This scale has demonstrated both reliability and concurrent validity (Conners et al., 2006). In this study, the scale demonstrated good reliability across time for both mothers (α= .82 to .87) and fathers (α= .80 to .83).

### Dependent Variable: Child Abuse Risk

Child abuse risk was assessed using two separate measures. First, parents reported on their own use of PCA with their child at both Times 3 (18 months old) and 4 (age 4) using the CTSPC (Straus et al., 1998), timepoints when the administration of the CTSPC would be appropriate. Comparable to the retrospective version above, participants indicated the frequency with which they used the 22 items with their child, with the same frequency count categories and weighted scoring, with each item again having a minimum score of 0 and a maximum score of 25. A higher CTSPC Combined Assault score summed across the 18 items involving physical and psychological PCA indicatse parents’ greater use of PCA.

Second, the *Response Analog to Child Compliance Task* (ReACCT; Rodriguez, 2016) is a computerized analog measure that reduces social desirability responding designed to indirectly assess the theoretical construct of child abuse risk which can be used prenatally. Administered at all four time points, the ReACCT measure simulates how parents manage child compliance or noncompliance. The respondent is asked to imagine they are the parent described in the ReACCT task, involving a realistic situation wherein the parent and child purportedly are late to get the child to school. Twelve sequential scenes are presented where the parent provides an instruction to the child to get them ready to leave home and the child is either compliant or noncompliant, possibly remaining remain stuck in one scene if the child remains noncompliant. Parents are asked to choose from 16 options for how they would respond to their child’s behavior, which include either adaptive responses (receiving positive weights) or maladaptive PCA responses (receiving negative weights). During the entire task, to induce time urgency, a visual clock counts time lapsed with an audio of a ticking clock; parents are told each time they secure quick compliance, they will receive a game bonus ($0.50). The current study focused on parents’ responses to the 12 noncompliance scenes, with scores ranging from − 36 to 60 and higher Noncompliance scores suggesting greater abuse risk. Across samples of varying risk, ReACCT Noncompliance scores have demonstrated concurrent and convergent validity with traditional child abuse risk measures as well as actual PCA use (Rodriguez, 2016; Rodriguez & Silvia, 2022). The current study demonstrated good reliability across time for mothers (α= .76 to .83) and fathers (α= .76 to .79).

### Data Analysis

#### Missing Data

Although attrition was minimal between T1 through T3, several families could not be located or were not locally available by the time the study was extended for T4. Thus, differential attrition across time was examined by testing whether those retained differed from those not retained on demographic variables or any of the outcome measures using *t*-tests or Chi-square analyses, as appropriate. Findings indicated that those not retained did not significantly differ from those retained across time on race, age, income, educational level, or outcome measures with the exception that between T3 and T4, those not retained obtained higher T3 ReACCT Noncompliance scores (*M*=2.58, *SD*=13.62) than those retained (*M*=−0.71,*SD*=13.89), *t*=2.01, *p*=.039, and males compared to females from T1 were less likely to be retained by T4, *χ*^2^=3.83, *p*=.05. Overall, there was minimal evidence of differential attrition.

#### Analyses

**Fig. 1:**
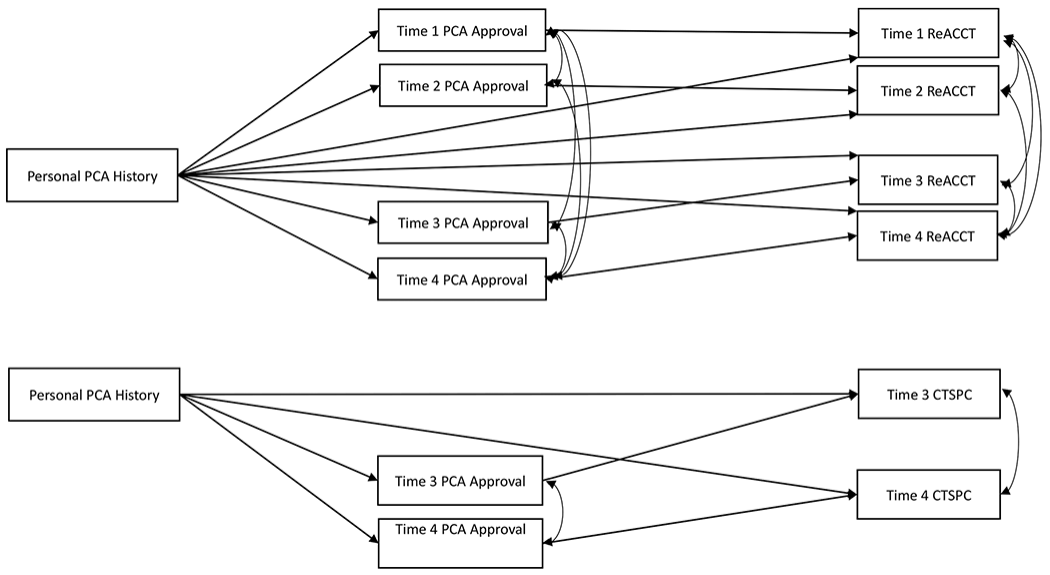
Mediation Conceptual Models. Note PCA=Parent-Child aggression; ReACCT=Response Analog to Child Compliance Task, Noncompliance scale. CTSPC=Parent-Child Conflict Tactics Scale, Combined Assault. The statistical model includes lagged coeffcients across time for the proposed mediator and similar lagged coeffcients for the outcome variables

Data reported in this study involved no manipulations or exclusions. Preliminary descriptive statistics and correlations were conducted in SPSS 27. Because income and educational level were significantly intercorrelated (e.g., *r*>.68 across time), these values were standardized and combined into a socioeconomic (SES) composite score. For the primary analyses, a multigroup mediation path analysis, which incorporates a test of whether paths differ by racial group, was performed to predict child abuse risk (ReACCT Noncompliance) at four time points from CTSPC Combined Assault History scores at T1 with PCA approval from AAPI-2 Corporal Punishment scores at each time point as partial mediators (see Fig. 1). Separately, given there were only two age-appropriate time points for PCA use, CTSPC Combined Assault History scores at T1 predicted their CTSPC Combined Assault scores at T3 and T4, with AAPI-2 Corporal Punishment Scores at T3 and T4 as partial mediators, also testing whether paths differ by racial group. These path analyses were tested with Mplus 8.1 using Full Information Maximum Likelihood (FIML) estimation to maximize use of all available data. All models included lagged paths of the proposed mediator and outcome variable (stability of that given variable across time) to accommodate covariance within the same construct at multiple times. Data were nested within families (e.g., mothers partnered with fathers) using the Cluster command in Mplus to avoid violating the independence assumption. Furthermore, the model Constraint command in Mplus constrained individual paths to be equal to test for path differences between racial groups and the Model Indirect Command tested for indirect effects of PCA approval. Acceptability of overall model fit was judged using root mean square error of approximation (RMSEA), standardized root-mean-square residual (SRMR), and comparative fit index (CFI), with RMSEA and SRMR values below .08 and CFI values above .95 considered acceptable (Kline, 2011). All path coefficients presented are standardized. Although significance at .05 was adopted for these analyses, potential marginal effects (*p*<.10) are noted given these highly controlled statistical models incorporate stability (lagged) estimates and covariates.

## Results

### Preliminary Analyses

Means, standard deviations, and bivariate correlations for all measures per group appear in Table 1. All findings provided in Table 1 do not account for the clustered nature of the data, do not reflect the covariances between constructs, and do not include important covariates, and should thus be interpreted with caution. Given apparent racial group differences in SES and age, both were used as covariates in the primary path analyses below. Note that personal PCA History was generally consistently related to PCA Approval (AAPI-2 Corporal Punishment scale) as well as ReACCT Noncompliance and CTSPC Combined Assault scores for Black parents but not for White parents. Some group differences were identified with t-tests in raw data, with significantly higher ReACCT Noncompliance scores observed for Black parents compared to White parents across time but no significant differences were noted for PCA history or PCA use scores. Black parents reported significantly stronger PCA approval than White parents only at Times 2 and 3.

### Path Analyses: ReACCT Noncompliance

For the analyses using ReACCT Noncompliance scores as the outcome measure, controlling for SES and age, the model demonstrated good fit, CFI=.97, RMSEA=.06, SRMR=.07. The path coefficients (see Table 2) indicate that for Non-Hispanic White parents, personal PCA History was significantly related to PCA Approval (H1a) only at T1 and T3, but at T3, *greater* personal PCA History was predictive of *lower* PCA Approval. Greater PCA Approval was significantly associated with higher ReACCT Noncompliance (H1b) at all timepoints except at T4, when the effect became marginal. Notably, greater personal PCA History demonstrated no direct effects on ReACCT Noncompliance at any time point for White parents (H1c). Significant indirect effects through PCA Approval (H2a) were observed at T1 and T3 but only at Time 1 was this indirect effect through *greater* PCA Approval (at T3, the indirect effect was negative).

For Black parents, greater personal PCA History was significantly related to stronger PCA Approval (H1a) at T1 and T4 and marginally at T3 in the expected direction; Black parents additionally retained a significant direct effect of more personal PCA History on higher ReACCT Noncompliance scores by T4 (H1c). Similar to non-Hispanic White parents, greater PCA Approval was related to higher ReACCT Noncompliance scores at T1-T3 (H1b); the effect was marginal at T4. Significant indirect effects from personal PCA History to higher ReACCT Noncompliance scores through greater PCA Approval were observed for Black parents at T1 only (H2a). Significant differences between racial groups (H1d) were identified for the effect between greater personal PCA History and PCA Approval at T3 (*p*=.001), the path between PCA Approval and ReACCT Noncompliance scores at Time 1 (*p*=.05), as well as a significant difference in the indirect effect (H2b) through PCA Approval at T3 (*p*=.03), likely given the directional differences regarding the effect of PCA Approval between racial groups.

### Path Analyses: CTSPC Combined Assault

For the analyses on parents’ PCA Use utilizing their T3 and T4 CTSPC Combined Assault scores as the outcome measure, controlling for SES and age, the model demonstrated acceptable fit, CFI=.96, RMSEA=.08, SRMR=.07. As seen in Table 2, for Non-Hispanic White parents, personal PCA History did not significantly predict PCA Approval at either time point (H1a). Greater PCA Approval significantly predicted parents’ more frequent PCA Use (higher CTSPC Combined Assault scores) at T3, but the effect became marginal at T4 (H1b). Personal PCA History was not significantly directly related to parents’ own PCA Use at T3 but was significantly related to their PCA Use at T4 (H1c). No indirect effects through greater PCA Approval were observed for Non-Hispanic White parents at either time point (H2a).

For Black parents, more frequent personal PCA History significantly predicted greater PCA Approval (H1a) at both T3 and T4. Stronger PCA Approval significantly predicted more frequent PCA Use (higher CTSPC Combined Assault scores) at both T3 and T4 (H1b). The direct effect of personal PCA History on Black parents’ use of PCA at T3 was significant but not by T4 (H1c). PCA Approval significantly partially mediated at T3 but only marginally by T4 (H2a). Significant racial group differences (H1d) were observed for the effect of personal PCA History on parents’ PCA Use at T3 (*p*=.006).

## Discussion

The current study used longitudinal data to investigate the role of PCA approval as a factor in intergenerational effects wherein the effects of personal PCA history on parents’ child abuse risk could differ by race, rather than assume that risk factors operate equivalently across groups. Two separate measures of child abuse risk (one an analog measure of parents’ risk broadly that could be assessed prenatally and another on parents’ actual PCA use) were used to determine the robustness of observed effects. Notably, personal PCA history predicted greater PCA approval (H1a) for Black parents on both measures of abuse risk. In contrast, these direct effects were mixed for White parents: either absent (with CTSPC Combined Assault) or a reverse effect (with ReACCT Noncompliance) of lower PCA approval predicting greater abuse risk for White parents. This effect of PCA history predicting greater PCA approval on both measures of abuse risk differed between groups (H1d). On both outcome measures and for both racial groups, greater PCA approval was associated with child abuse risk (H1b). Personal PCA history only significantly predicted actual PCA use (CTSPC) at the final timepoint for White parents; for Black parents, personal history predicted theoretical abuse risk (ReACCT) at the last time point and actual PCA use at the third time point (H1c). Whereas PCA approval demonstrated mediation for Black parents, such mediation was only apparent for White parents on the broad measure of abuse risk assessed prenatally, with evident group differences (H1d). Such findings suggest contributors to intergenerational processes may differ by race which could be better considered when creating more culturally appropriate prevention programs rather than assuming all risk factors affect all parents equally.

### Direct Effects of PCA History on PCA Approval

Based on previous literature, personal history of PCA was hypothesized to significantly relate to PCA approval (Durrant et al., 2018; Rodriguez et al., 2018b). The connection between PCA history and PCA approval differed between White and Black parents. For Non-Hispanic White parents, during the last trimester of pregnancy, greater history of PCA related to PCA approval. Yet by the time their toddler was 18 months, contrary to expectations, the findings were mixed, with greater personal history of PCA predictive of *lower* PCA approval for White parents on the measure of theoretical child abuse risk and unrelated to reported PCA use. This finding could reflect differences in parental beliefs that evolve over time and that are impacted by cultural influences (Lansford et al., 2017; Lansford, 2022). Expectant Non-Hispanic White parents may initially approve of PCA based on their personal history of PCA—aligning with their own history without experience of children of their own— but appear to shift their attitudes once they transition into parenthood and directly interact with their own child entering toddlerhood. Such a shift warrants additional exploration. However, for Black parents, as hypothesized, greater personal history of PCA was related to their approval of PCA except when their child was an infant on one of the two outcome measures. Overall, the link with personal PCA history and PCA approval presented in the expected direction for Black parents relative to White parents, which may account for the observed racial differences in PCA (Gershoff et al., 2012; Finkelhor et al., 2019; Taillieu et al., 2014) and PCA approval (Chiocca, 2017; Su et al., 2018) previously reported in the literature.

### Direct Effects of PCA History on Abuse Risk

Personal PCA history was also expected to directly predict child abuse risk, with greater personal history of physical and psychological PCA increasing parents’ risk of abuse with their own children. Contrary to previous literature on the intergenerational effects of personal history and abuse risk (Bartlett et al., 2017; Buisman et al., 2020; Greene et al., 2020), greater personal history for White parents did not significantly predict their abuse risk except for one effect at the final time point with the measure of PCA use. These findings align with some discussion of the potential artifact of much of the existing literature being based on cross-sectional rather than longitudinal designs (see Rodriguez et al., 2018b for discussion; Widom et al., 2015). Black parents’ PCA personal history significantly predicted higher abuse risk on the broader measure at the final timepoint. Black parents’ PCA personal history also significantly predicted PCA use during toddlerhood, which further differed between racial groups. Such differences are consistent with observed racial differences in parental child abuse risk in the U.S. (Rodriguez et al., 2021; Silveira et al., 2021). Intergenerational effects on PCA approval and PCA use appear more consistently for Black parents compared to White parents, suggesting a greater tendency for Black parents to adopt the same PCA approaches they experienced as children. As has been suggested previously (Patton, 2017; Silveira et al., 2021), Black parents may align more strongly with their heritage and evidence more intergenerational continuity, implementing PCA from a protective stance toward their child in anticipation of physical violence being perpetrated by the community against Black youth (Patton et al., 2021).

### Direct Effects of PCA Approval on Abuse Risk

As hypothesized, greater PCA approval was significantly associated with higher abuse risk for both White and Black parents. These findings support recent data demonstrating that PCA approval contributes to parents’ physical abuse risk (Camilo et al., 2020; Lansford et al., 2014) and both physical and psychological aggression (Combs-Orme & Cain, 2008; Rodriguez et al., 2023). Thus, reducing approval of PCA could impact all parents’ child abuse risk and should feature prominently in abuse prevention programs.

### Indirect Effects Between PCA History and Abuse Risk Through PCA Approval

Findings from the present study support our hypothesis that PCA approval can mediate the relation between personal PCA history and abuse risk, with somewhat more evident effects for Black parents than White parents. More specifically, the indirect effects for the broader measure of abuse risk were comparable between the two racial groups during pregnancy in the hypothesized direction; however, in toddlerhood, the indirect effects were in the reverse direction for White parents. Potentially due to the strong direct effects of Black parents’ personal history on abuse risk, indirect effects were only evident during pregnancy—before having their own child, when they may be more strongly influenced by how they were parented—but not when the child was age 4 years. Such findings highlight the relevance and strength of personal history on abuse risk for Black parents, even when considering the role of PCA approval. Additionally, no indirect effects from personal history through greater PCA approval were observed for White parents regarding PCA use but were observed for Black parents during toddlerhood—a developmental period when behavior problems may become more challenging. Thus, difference in effects by developmental period may reflect parents’ adjustment in parenting strategies to child behavior and temperament as the child ages as parents begin to consider the need to socialize their children to societal threats (Patton, 2017; Silveira et al., 2021). Overall, personal history appears to exert more influence both directly and indirectly through PCA approval for Black parents, suggesting again the significance of cultural heritage for Black families identified in previous literature (Patton, 2017; Silveira et al., 2021). Thus, these indirect effects suggest that adapting targeted, developmental stage-based interventions to reduce PCA approval in a culturally sensitive framework could better engage clients to facilitate breaking intergenerational influences. Further, although PCA approval may perpetuate the abuse cycle, additional unidentified factors may also contribute to the effects of personal history on abuse risk.

### Limitations and Future Research Directions

Current results should be interpreted in the context of some study limitations, which can help guide future research. Parent-child relationships and interactions evolve based on a child’s developmental stage. Inconsistencies in findings across time may reflect the evolving challenges parents face across the transition into parenthood. As an example, research has shown that parental perspectives on children’s problem behavior and infant temperament can influence their PCA use (Rodriguez & Wittig, 2019). Although children under three represents the highest risk for maltreatment (DHHS, 2022), future research could extend data collection to older children (e.g., school-aged or adolescent) to evaluate trajectories in PCA approval as children age and parenting demands continue to shift. Given that the potential intergenerational effects of personal PCA history may evolve over time, continued work with longitudinal studies with longer retention and additional time points is needed. Furthermore, although retention was strong in the initial phases of the study, more attrition occurred during the unplanned extension of the study.

Although history theoretically predates the other constructs, personal history was assessed during the first time point concurrently with the mediator and outcomes for the first wave (although assessed before all variables in later timepoints). Ideally, history would be assessed prior to other variables even at the first wave. Assessing the mediator at an earlier timepoint than the outcomes should also be investigated. Currently, given that our analyses involve the mediator and proposed outcomes evaluated at the same timepoint, abuse risk could have been analyzed as the mediator and PCA approval as the outcome (personal history → abuse risk → PCA approval). Additional analyses probing such alternative models revealed similar patterns and no differences in indirect effects given the observed bidirectional relations between variables. Our analyzed models are consistent with current theory and meta-analyses (Camilo et al., 2020), but future research could examine alternative statistical models of these factors across time. Additionally, current findings are based on retrospective self-reports of PCA personal history, which can be limited methodologically. Future studies should consider alternative strategies to gather personal history of PCA that may be able to ascertain the veracity of such reports (e.g., obtaining corroborated reports through DHHS). The present findings also include parents’ self-reports of their own PCA approval and PCA use, which are subject to social desirability biases. Although the current design used two outcome measures to probe the robustness of effects—including an analog assessment of child abuse risk to reduce such social desirability concerns—future research should consider additional, multi-method approaches across domains of interest. Some of the inconsistencies in our results may be attributable to our effort to increase the robustness of findings using a traditional self-report that is vulnerable to social desirability as well as an analog measure. Interpretations should consider the nuances of the measure administered and the potential social desirability bias impacting that outcome, especially within the context of the CTSPC. Moreover, a larger sample would be able to add paths to test how a potential mediator—such as PCA approval at one point—can predict later abuse risk. Indeed, apart from PCA approval, other individual factors not examined in the present study can influence abuse risk (e.g., personality, temperament, caregiving role, etc.) which could also be considered in future research.

A particularly interesting line of research could consider how gender and race intersect to evaluate how mothers and fathers within racial groups adopt and enact beliefs differently that impact their abuse risk—a feature we could not address with our sample size. Moreover, we observed substantial differences in SES between groups, which we controlled for in our main analyses; but recent research has also shown the impact of lower educational attainment on corporal punishment and PCA use in White families in particular (Schneider & Schenck-Fontaine, 2022) which suggests further investigation into the effects of SES in this subgroup. A final limitation relates to the composition of the sample: self-identified Non-Hispanic, non-biracial White parents, or self-identified Black parents. Future research should examine the nuances of multiracial identities and other intersecting identities, which may also affect personal history of PCA, PCA approval, and/or parents’ child abuse risk.

### Prevention and Clinical Implications

This study used longitudinal data to address an important gap in the literature by examining what may contribute to racial differences in the intergenerational cycle of child abuse risk. Meta-analyses suggest that current child abuse prevention programs exert modest effects at best, with weaker effects for parents of color (van der Put et al., 2018), underscoring the need for interventions that are more culturally relevant to the parents they serve. The current study findings introduce evidence to suggest that not all child abuse risk factors are universally applicable. PCA approval does appear consistently related to child abuse risk across groups. Thus, future abuse prevention programs must consider ways in which to target both attitudes about PCA and PCA behaviors. Goals would include reducing both approval and use of PCA in all parents by engaging in cognitive reframing, teaching alternative forms of discipline, and guidance on culturally appropriate conflict resolution strategies.

Our results suggest a more consistent relevance of personal history for Black parents in their risk for engaging in physical and psychological aggression, and in the mediating role of PCA approval, compared to White parents. Yet, with both groups, the findings suggest researchers and interventionists must work more diligently to develop culturally appropriate programming (e.g., Mejia et al., 2017; Nystrand et al., 2021; Osman et al., 2022) that moves parents beyond their personal history. Culturally tailored programming could include special attention to the language parents use, exploring the origins of parenting or discipline beliefs, and adapting resources to cultural and societal contexts (see Osman et al., 2022). Moreover, the findings bolster the importance of intervening at multiple points across developmental stages. For example, research has shown that mothers with less knowledge of non-physical discipline options during infancy later reported increased problem behaviors in their toddlers (Rodriguez & Wittig, 2019). Therefore, intervening during infancy and targeting parents with harsher PCA histories could help minimize abuse risk during infancy, early childhood, and toddlerhood by improving knowledge of alternative discipline approaches. Additionally, adding brief interventions through toddlerhood—a developmental period that may pose new challenges for parents—could help parents recognize problem behaviors and learn skills to manage them.

Acknowledging the historical role of PCA use in Black families should be approached with respectful cultural humility, recognizing the realities of systemic sociocultural inequities that may motivate Black parents to implement PCA to protect their children (Patton, 2017; Silveira et al., 2021). Given the differences in lived experiences, an important target involves clinicians partnering with Black parents to incorporate background on how the historical roots of PCA derive from systems that were designed to oppress communities of color (Patton, 2017). Relatedly, racially-driven SES disparities and systemic inequities need to be appreciated when preparing prevention and intervention efforts. Future research should identify alternative mechanisms for how personal history affects abuse risk for Black parents, as well as how protective resources may mitigate those effects, which can further guide the development of more culturally informed approaches to child abuse prevention programs. The current study begins to clarify potential racialized components in parenting that may help maintain or disrupt the child abuse cycle. In so doing, we aimed to recognize when risk factors may not operate universally by moving away from considering race as an explanatory factor in child abuse risk.

## Figures and Tables

**Table 1:** Means, Standard Deviations, Correlations, and t-tests by Group

	1	2	3	4	5	6	7	8	9	10	11
1. PCA History		.37***	.24**	.29***	.19*	.29***	.20*	.51***	.30***	.30**	.46***
2. T1 PCA Approval	.18*		.48***	.67***	.40***	.53***	.23**	.33***	.50***	.33**	.31**
3. T1 ReACCT	.15*	.57***		.43***	.66***	.40***	.57***	.29***	.27*	.47***	.19
4. T2 PCA Approval	.12	.81***	.52***		.45***	.65***	.23**	.30***	.63***	.25*	.37***
5. T2 ReAACT	.09	.48***	.65***	.53***		.41***	.57***	.27**	.35**	.56***	.23*
6. T3 PCA Approval	.03	.74***	.56***	.79***	.60***		.43***	.37**	.57***	.33**	.34**
7. T3 ReAACT	.20*	.48***	.58***	.47***	.73***	.53***		.27***	.20	.61***	.20
8. T3 CTSPC	.09	.34***	.40***	.31***	.43***	.45***	.37***		.50***	.34**	.77***
9. T4 PCA Approval	.07	.72***	.54***	.73***	.51***	.83***	.50***	.40***		.37***	.52***
10.T4 ReACCT	.19	.42***	.64***	.37***	.65***	.49***	.64***	.41***	.53***		.38***
11. T4 CTSPC	.17	.18	.30**	.21*	.27**	.28**	.32**	.47***	.40***	.40***	
**White M**	73.34	31.44	−3.43	29.51	−3.63	28.64	−3.66	12.71	28.78	−6.39	26.81
**White SD**	63.79	9.49	10.91	9.56	12.10	9.99	11.90	17.25	10.11	10.39	25.57
**Black M**	84.37	32.80	3.76	31.50	5.22	31.36	5.26	14.45	30.75	−2.25	20.50
**Black SD**	81.40	7.57	13.60	7.84	14.67	7.79	14.31	23.54	8.78	13.04	28.19
t-values	−1.36	−1.43	−5.26***	−2.01*	−5.70***	−2.66**	−5.92***	−0.72	−1.40	−2.35**	1.56

Note White parents below the diagonal, Black parents above the diagonal. PCA=Parent-child aggression; T1: Time 1 (prenatal); T2: Time 2 (6 months); T3: Time 3 (18 months); T4: Time 4 (4 years). ReACCT=Response Analog to Child Compliance Task Noncompliance. CTSPC=Parent-Child Conflict Tactics Scale Combined Assault

* *p* ≤.05,

** *p* ≤.01,

*** *p* ≤.001

**Table 2:** Standardized coefficients for groups in path models separately by outcome measure

Parameter estimate	White			Black		
	
*ReACCT: Direct Effects*	β	*p*	95% CI	β	*p*	95% CI
PCA History → T1 PCA Approval	.20	.015	.07, .42	.40	<.001	.29, .59
PCA History → T2 PCA Approval	−.06	.188	−.12, .05	.03	.693	−.09, .22
PCA History → T3 PCA Approval	**−.13**	**.003**	−.21, −.02	.11	.065*	.01, .27
PCA History → T4 PCA Approval	.06	.232	−.02, .19	**.18**	**.024**	.05, .38
T1 PCA Approval → T1 ReACCT	**.55**	**<.001**	.47, .67	**.49**	<.001*	.39, .64
T2 PCA Approval → T2 ReACCT	**.29**	**<.001**	.19, .44	**.24**	**<.001**	.13, .41
T3 PCA Approval → T3 ReACCT	**.13**	**.023**	.04, .28	**.19**	**.017**	.06, .39
T4 PCA Approval → T4 ReACCT	.14	.080	.01, .34	.16	.086	.01, .40
PCA History → T1 ReACCT	.04	.445	−.04, .17	.09	.184	−.02, .26
PCA History → T2 ReACCT	−.02	.743	−.10, .12	.02	.820	−.11, .21
PCA History → T3 ReACCT	.10	.137	−.01, .26	.08	.348	−.06, .28
PCA History → T4 ReACCT	.06	.263	−.03, .18	**.21**	**.022**	.06, .44
***ReACCT Indirect Effects***	**β**	***p***	**95% CI**	**β**	***p***	**95% CI**
PCA History → T1 PCA Approval → T1 ReACCT	**.11**	**.019**	.03, .23	**.20**	**<.001**	.13, .31
PCA History → T2 PCA Approval → T2 ReACCT	−.02	.210	−.04, .02	.01	.700	−.02, .05
PCA History → T3 PCA Approval → T3 ReACCT	**−.02**	**.035**	−.03, −.00	.02	.168*	−.00, .06
PCA History → T4 PCA Approval → T4 ReACCT	.01	.313	−.01, .03	.03	.158	−.01, .08
***CTSPC Direct Effects***	**β**	***p***	**95% CI**	**β**	***p***	**95% CI**
PCA History → T3 PCA Approval	.03	.758	−.13, .29	**.29**	**<.001**	.18, .46
PCA History → T4 PCA Approval	.08	.127	−.01, .23	**.21**	**.036**	.05, .46
T3 PCA Approval → T3 CTSPC	**.42**	**<.001**	.30, .59	**.24**	**<.001**	.15, .39
T4 PCA Approval → T4 CTSPC	.20	.061	.02, .47	**.24**	**<.001**	.14, .40
PCA History → T3 CTSPC	.08	.245	−.04, .27	**.40**	<.001*	.25, .65
PCA History → T4 CTSPC	**.17**	**.035**	.04, .38	.02	.852	−.13, .24
***CTSPC Indirect Effects***	**β**	***p***	**95% CI**	**β**	***p***	**95% CI**
PCA History → T3 PCA Approval → T3 CTSPC	.01	.758	−.06, .12	**.07**	**.003**	.03, .13
PCA History → T4 PCA Approval → T4 CTSPC	.02	.218	−.01, .05	.05	.091	.00, .13

Note SES and age included as covariates. T1: Time 1 (prenatal); T2: Time 2 (6 months); T3: Time 3 (18 months); T4: Time 4 (4 years). PCA=Parent Child Aggression. ReACCT=Response Analog to Child Compliance Task, Noncompliance scale. CTSPC=Parent-Child Conflict Tactics Scale, Combined Assault. **Bolded** values indicate statistical significance. Significant differences between groups are signified with an asterisk. Covariances and lagged estimates available upon request

## Data Availability

Upon request.
